# (4-Fluoro­phen­yl-κ*C*)(*N*,*N*,*N*′,*N*′-tetra­methyl­ethylenedi­amine-κ^2^ *N*,*N*′)(tri­fluoro­meth­yl-κ*C*)palladium(II)

**DOI:** 10.1107/S1600536814007855

**Published:** 2014-04-16

**Authors:** Youzhi Du, ChangGe Zheng

**Affiliations:** a School of Chemical and Material Engineering, Jiangnan University, 1800 Lihu Road, Wuxi, Jiangsu Province 214122, People’s Republic of China

## Abstract

In the title compound, [Pd(CF_3_)(C_6_H_4_F)(C_6_H_16_N_2_)], the Pd^II^ cation is four-coordinated by the two N atoms of the *N*,*N*,*N*′,*N*′-tetra­methyl­ethylenedi­amine ligand and by one C atom each from a 4-fluoro­phenyl and a tri­fluoro­methyl ligand, in a distorted rectangular-planar geometry, with an average deviation from the least-squares plane of 0.066 (2) Å. The central coordination angles with the Pd^II^ atom range from 83.14 (10) to 97.25 (12)°.

## Related literature   

For metal-mediated C—F bond-breaking and C—C bond-formation reactions in similar compounds, see: Maleckis & Sanford (2011 ▶); Ball *et al.* (2010 ▶, 2011 ▶); Ye *et al.* (2010 ▶); Racowski *et al.* (2011 ▶). For similar Pd^II^—CF_3_ bonds, see: Grushin & Marshall (2006 ▶).
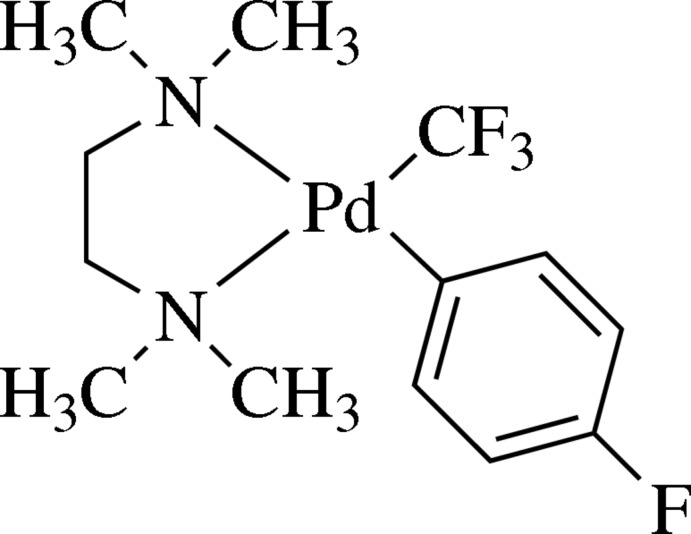


## Experimental   

### 

#### Crystal data  

[Pd(CF_3_)(C_6_H_4_F)(C_6_H_8_N_2_)]
*M* *_r_* = 386.71
Monoclinic, 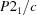

*a* = 16.6651 (19) Å
*b* = 8.3464 (9) Å
*c* = 11.4710 (13) Å
β = 103.063 (2)°
*V* = 1554.3 (3) Å^3^
*Z* = 4
Mo *K*α radiation
μ = 1.23 mm^−1^
*T* = 296 K
0.29 × 0.27 × 0.19 mm

#### Data collection  

Bruker APEXII CCD diffractometer
Absorption correction: multi-scan (*SADABS*; Bruker, 2007 ▶) *T*_min_ = 0.717, *T*_max_ = 0.800
8555 measured reflections
2875 independent reflections
2628 reflections with *I* > 2σ(*I*)
*R*_int_ = 0.032

#### Refinement  

*R*[*F* ^2^ > 2σ(*F* ^2^)] = 0.026
*wR*(*F* ^2^) = 0.067
*S* = 1.04
2875 reflections
180 parameters
H-atom parameters constrained
Δρ_max_ = 0.56 e Å^−3^
Δρ_min_ = −0.42 e Å^−3^

### 

Data collection: *APEX2* (Bruker, 2007 ▶); cell refinement: *SAINT* (Bruker, 2007 ▶); data reduction: *SAINT*; program(s) used to solve structure: *SHELXS97* (Sheldrick, 2008 ▶); program(s) used to refine structure: *SHELXL97* (Sheldrick, 2008 ▶); molecular graphics: *SHELXTL* (Sheldrick, 2008 ▶); software used to prepare material for publication: *SHELXTL*.

## Supplementary Material

Crystal structure: contains datablock(s) I, New_Global_Publ_Block. DOI: 10.1107/S1600536814007855/vn2082sup1.cif

Structure factors: contains datablock(s) I. DOI: 10.1107/S1600536814007855/vn2082Isup2.hkl

CCDC reference: 996158

Additional supporting information:  crystallographic information; 3D view; checkCIF report

## Figures and Tables

**Table 1:** Selected bond lengths (Å)

Pd1—C12	2.004 (3)
Pd1—C13	2.017 (3)
Pd1—N1	2.172 (2)
Pd1—N2	2.206 (2)

**Fig. 1.**
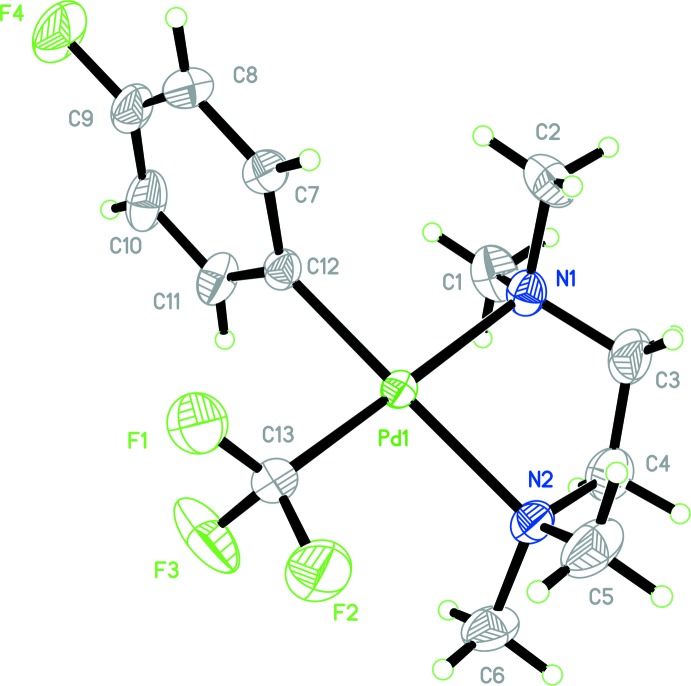
A view of the molecular structure of [(tmeda)Pd(p-FPh)(CF3)] with displacement ellipsoids drawn at the 30% probability level.

**Fig. 2.**
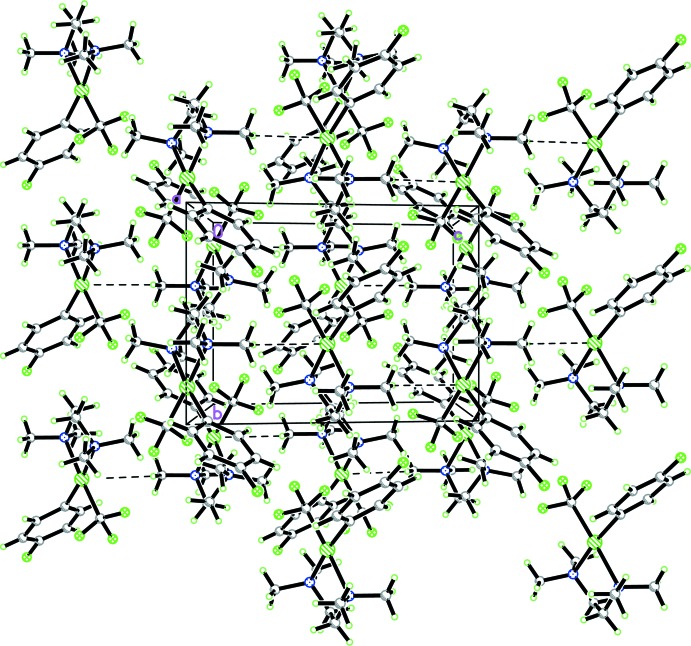
The molecular packing of [(tmeda)Pd(p-FPh)(CF3)], viewed along the a axis. Atom codes: Pd checkered green spheres, F hatched green spheres, N blue dotted spheres, C black spheres, H green circled spheres.

## supplementary crystallographic information

### 1. Comment

Trifluoromethyl palladium aryl complexes have attracted much attention due to the demand for metal-mediated C—F bond breaking and C—C bond formation reactions involving highly fluorinated substrates (Maleckis & Sanford, 2011; Ball *et al.*, 2010; Ye *et al.*, 2010; Racowski *et al.*, 2011; Ball *et al.*,2011).

Single-crystal X-ray diffraction of the title compound reveals that Pd^II^ [(tmeda)Pd(*p*-FPh)(CF_3_)] is four-coordinate. As shown in Fig. 1, the asymmetric unit comprises one Pd^II^ cation, a tmeda ligand (N1 and N2), a *p*-FPh group (C12) and a CF_3_ group (C13). For primary bond lengths, see Table 1. The Pd^II^—CF_3_ bond length [2.015 (4) Å] (Table 1) is comparable with those in similar Pd^II^ complexes (Grushin & Marshall, 2006). The bidentate Xantphos ligand used in the latte study is s a larger spatial stucture ligand, and therefore [(Xantphos)Pd(Ph)(CF_3_)] has an obviously greater Pd^II^—CF_3_ bond length with [2.069 (3) Å] (Grushin & Marshall, 2006). Fig. 2 gives the molecular packing of the title compound, viewed along the *a* axis.

### 2. Experimental

Under nitrogen, [(tmeda)Pd(*p*-FPh)(I)] (445 mg, 1 mmol, 1 equiv) and CsF (3 equiv) were suspended in THF (0.145 *M*) in a 25 ml Schlenk flask. This mixture was stirred strongly for 10 min and then TMSCF_3_ (2 equiv) was added. The reaction was stirred vigorously for 1.5 h at 30 °C. A colorless solid [(tmeda)Pd(*p*-FPh)(CF_3_)] was obtained. 50 mg of [(tmeda)Pd(*p*-FPh)(CF_3_)] was put into a 10 ml transparent bottle, and CH_2_Cl_2_ (2 ml) was added to dissolve it. The bottleneck was sealed by a plastic wrap, and lay aside the transparent bottle into a wide-mouth bottle containing diethyl ether (15 ml). Colorless block single crystals of [(tmeda)Pd(*p*-FPh)(CF_3_)] were obtained after 3 days.

### 3. Refinement

The H atoms bonded to C atoms were introduced at calculated positions and refined using a riding model, with *U*_iso_(H) = 1.2*U*_eq_(C) and *U*_iso_(H) = 1.5*U*_eq_(C), and C–H distances of 0.93–0.96 Å.

### Crystal data

**Table:**

[Pd(CF_3_)(C_6_H_4_F)(C_6_H_16_N_2_)]	*F*(000) = 776
*M**_r_* = 386.71	*D*_x_ = 1.653 Mg m^−^^3^
Monoclinic, *P*2_1_/*c*	Mo *K*α radiation, λ = 0.71073 Å
Hall symbol: -P 2ybc	Cell parameters from 6327 reflections
*a* = 16.6651 (19) Å	θ = 2.5–28.3°
*b* = 8.3464 (9) Å	µ = 1.23 mm^−^^1^
*c* = 11.4710 (13) Å	*T* = 296 K
β = 103.063 (2)°	Block, colourless
*V* = 1554.3 (3) Å^3^	0.29 × 0.27 × 0.19 mm
*Z* = 4	

### Data collection

**Table:**

Bruker APEXII CCD diffractometer	2875 independent reflections
Radiation source: fine-focus sealed tube	2628 reflections with *I* > 2σ(*I*)
Graphite monochromator	*R*_int_ = 0.032
φ and ω scans	θ_max_ = 25.5°, θ_min_ = 2.5°
Absorption correction: multi-scan (*SADABS*; Bruker, 2007)	*h* = −20→19
*T*_min_ = 0.717, *T*_max_ = 0.800	*k* = −7→10
8555 measured reflections	*l* = −13→13

### Refinement

**Table:**

Refinement on *F*^2^	Secondary atom site location: difference Fourier map
Least-squares matrix: full	Hydrogen site location: inferred from neighbouring sites
*R*[*F*^2^ > 2σ(*F*^2^)] = 0.026	H-atom parameters constrained
*wR*(*F*^2^) = 0.067	*w* = 1/[σ^2^(*F*_o_^2^) + (0.0257*P*)^2^ + 1.3072*P*] where *P* = (*F*_o_^2^ + 2*F*_c_^2^)/3
*S* = 1.04	(Δ/σ)_max_ < 0.001
2875 reflections	Δρ_max_ = 0.56 e Å^−^^3^
180 parameters	Δρ_min_ = −0.42 e Å^−^^3^
0 restraints	Extinction correction: *SHELXL97* (Sheldrick, 2008), Fc^*^=kFc[1+0.001xFc^2^λ^3^/sin(2θ)]^-1/4^
Primary atom site location: structure-invariant direct methods	Extinction coefficient: 0.0130 (7)

### Special details

**Table:**

Geometry. All e.s.d.'s (except the e.s.d. in the dihedral angle between two l.s. planes) are estimated using the full covariance matrix. The cell e.s.d.'s are taken into account individually in the estimation of e.s.d.'s in distances, angles and torsion angles; correlations between e.s.d.'s in cell parameters are only used when they are defined by crystal symmetry. An approximate (isotropic) treatment of cell e.s.d.'s is used for estimating e.s.d.'s involving l.s. planes.
Refinement. Refinement of *F*^2^ against ALL reflections. The weighted *R*-factor *wR* and goodness of fit *S* are based on *F*^2^, conventional *R*-factors *R* are based on *F*, with *F* set to zero for negative *F*^2^. The threshold expression of *F*^2^ > σ(*F*^2^) is used only for calculating *R*-factors(gt) *etc*. and is not relevant to the choice of reflections for refinement. *R*-factors based on *F*^2^ are statistically about twice as large as those based on *F*, and *R*- factors based on ALL data will be even larger.

### Fractional atomic coordinates and isotropic or equivalent isotropic displacement parameters (Å^2^)

**Table:**

	*x*	*y*	*z*	*U*_iso_*/*U*_eq_	
C1	0.1947 (3)	0.3541 (4)	−0.1824 (3)	0.0676 (10)	
H1A	0.1625	0.2614	−0.2124	0.101*	
H1B	0.2458	0.3510	−0.2077	0.101*	
H1C	0.1649	0.4492	−0.2130	0.101*	
C2	0.1308 (2)	0.3660 (4)	−0.0178 (4)	0.0697 (10)	
H2A	0.1389	0.3639	0.0677	0.104*	
H2B	0.0969	0.2770	−0.0517	0.104*	
H2C	0.1041	0.4643	−0.0482	0.104*	
C3	0.2600 (3)	0.4968 (4)	−0.0004 (4)	0.0770 (12)	
H3A	0.2441	0.5870	−0.0539	0.092*	
H3B	0.2472	0.5237	0.0757	0.092*	
C4	0.3477 (2)	0.4711 (5)	0.0176 (5)	0.0831 (13)	
H4A	0.3763	0.5657	0.0548	0.100*	
H4B	0.3611	0.4580	−0.0599	0.100*	
C5	0.3906 (3)	0.3625 (5)	0.2209 (4)	0.0832 (14)	
H5A	0.4169	0.2726	0.2661	0.125*	
H5B	0.3382	0.3815	0.2399	0.125*	
H5C	0.4247	0.4558	0.2406	0.125*	
C6	0.4590 (2)	0.2862 (5)	0.0683 (4)	0.0691 (10)	
H6A	0.4948	0.3776	0.0825	0.104*	
H6B	0.4514	0.2533	−0.0137	0.104*	
H6C	0.4831	0.2000	0.1199	0.104*	
C7	0.12779 (18)	−0.0466 (4)	−0.0076 (3)	0.0492 (7)	
H7	0.1232	−0.0043	0.0656	0.059*	
C8	0.0662 (2)	−0.1472 (4)	−0.0689 (4)	0.0658 (10)	
H8	0.0208	−0.1718	−0.0378	0.079*	
C9	0.0738 (2)	−0.2090 (4)	−0.1757 (4)	0.0658 (10)	
C10	0.1397 (3)	−0.1777 (4)	−0.2232 (3)	0.0675 (11)	
H10	0.1438	−0.2216	−0.2961	0.081*	
C11	0.2004 (2)	−0.0786 (4)	−0.1598 (3)	0.0557 (8)	
H11	0.2465	−0.0585	−0.1904	0.067*	
C12	0.19541 (17)	−0.0076 (3)	−0.0519 (2)	0.0389 (6)	
C13	0.34552 (19)	−0.0443 (4)	0.1069 (3)	0.056	
F1	0.30400 (16)	−0.1597 (3)	0.1466 (3)	0.0905 (8)	
F2	0.40331 (16)	−0.0049 (3)	0.2069 (3)	0.1081 (10)	
F3	0.3877 (2)	−0.1208 (3)	0.0386 (3)	0.1207 (12)	
F4	0.01374 (16)	−0.3096 (3)	−0.2362 (3)	0.1088 (10)	
N1	0.21161 (16)	0.3549 (3)	−0.0509 (2)	0.0455 (6)	
N2	0.37856 (16)	0.3287 (3)	0.0931 (2)	0.0483 (6)	
Pd1	0.282550 (12)	0.14760 (2)	0.027333 (17)	0.03517 (11)	

### Atomic displacement parameters (Å^2^)

**Table:**

	*U*^11^	*U*^22^	*U*^33^	*U*^12^	*U*^13^	*U*^23^
C1	0.092 (3)	0.062 (2)	0.0461 (18)	0.0159 (19)	0.0097 (18)	0.0128 (15)
C2	0.070 (2)	0.062 (2)	0.082 (3)	0.0226 (18)	0.027 (2)	0.0115 (19)
C3	0.080 (3)	0.0372 (19)	0.101 (3)	−0.0030 (18)	−0.006 (2)	0.0041 (19)
C4	0.071 (3)	0.045 (2)	0.132 (4)	−0.0135 (18)	0.019 (2)	0.009 (2)
C5	0.076 (3)	0.109 (4)	0.067 (2)	−0.034 (2)	0.022 (2)	−0.039 (2)
C6	0.0481 (19)	0.087 (3)	0.075 (2)	−0.0154 (19)	0.0203 (17)	−0.011 (2)
C7	0.0449 (16)	0.0494 (18)	0.0536 (17)	−0.0037 (14)	0.0119 (13)	−0.0032 (14)
C8	0.0386 (17)	0.060 (2)	0.097 (3)	−0.0064 (15)	0.0099 (18)	0.0007 (19)
C9	0.056 (2)	0.0447 (19)	0.079 (2)	−0.0073 (16)	−0.0217 (19)	−0.0024 (18)
C10	0.099 (3)	0.0480 (19)	0.0467 (18)	−0.0103 (19)	−0.0019 (19)	−0.0066 (15)
C11	0.074 (2)	0.0457 (18)	0.0504 (17)	−0.0156 (16)	0.0212 (16)	−0.0048 (15)
C12	0.0413 (14)	0.0342 (14)	0.0388 (13)	−0.0015 (11)	0.0038 (11)	0.0021 (11)
C13	0.044	0.049	0.072	−0.003	0.008	−0.006
F1	0.0801 (16)	0.0650 (15)	0.118 (2)	0.0029 (11)	0.0049 (14)	0.0432 (13)
F2	0.0912 (18)	0.0810 (17)	0.119 (2)	0.0006 (14)	−0.0460 (16)	0.0218 (15)
F3	0.129 (2)	0.098 (2)	0.154 (3)	0.0678 (19)	0.071 (2)	0.0274 (19)
F4	0.0831 (17)	0.0773 (16)	0.135 (2)	−0.0264 (14)	−0.0406 (16)	−0.0194 (16)
N1	0.0537 (15)	0.0382 (14)	0.0420 (13)	0.0000 (10)	0.0050 (11)	0.0040 (10)
N2	0.0434 (13)	0.0466 (14)	0.0544 (15)	−0.0089 (11)	0.0100 (11)	−0.0056 (12)
Pd1	0.03454 (14)	0.03201 (14)	0.03823 (14)	−0.00229 (8)	0.00669 (9)	−0.00038 (8)

### Geometric parameters (Å, º)

**Table:**

C1—N1	1.471 (4)	C6—H6B	0.9600
C1—H1A	0.9600	C6—H6C	0.9600
C1—H1B	0.9600	C7—C12	1.375 (4)
C1—H1C	0.9600	C7—C8	1.388 (5)
C2—N1	1.483 (5)	C7—H7	0.9300
C2—H2A	0.9600	C8—C9	1.361 (6)
C2—H2B	0.9600	C8—H8	0.9300
C2—H2C	0.9600	C9—C10	1.357 (6)
C3—C4	1.446 (6)	C9—F4	1.369 (4)
C3—N1	1.475 (4)	C10—C11	1.379 (5)
C3—H3A	0.9700	C10—H10	0.9300
C3—H3B	0.9700	C11—C12	1.392 (4)
C4—N2	1.492 (5)	C11—H11	0.9300
C4—H4A	0.9700	Pd1—C12	2.004 (3)
C4—H4B	0.9700	C13—F1	1.325 (4)
C5—N2	1.462 (5)	C13—F3	1.329 (4)
C5—H5A	0.9600	C13—F2	1.361 (4)
C5—H5B	0.9600	Pd1—C13	2.017 (3)
C5—H5C	0.9600	Pd1—N1	2.172 (2)
C6—N2	1.475 (4)	Pd1—N2	2.206 (2)
C6—H6A	0.9600		
			
N1—C1—H1A	109.5	C9—C8—C7	118.5 (4)
N1—C1—H1B	109.5	C9—C8—H8	120.8
H1A—C1—H1B	109.5	C7—C8—H8	120.8
N1—C1—H1C	109.5	C10—C9—C8	122.4 (3)
H1A—C1—H1C	109.5	C10—C9—F4	118.7 (4)
H1B—C1—H1C	109.5	C8—C9—F4	118.9 (4)
N1—C2—H2A	109.5	C9—C10—C11	117.9 (3)
N1—C2—H2B	109.5	C9—C10—H10	121.1
H2A—C2—H2B	109.5	C11—C10—H10	121.1
N1—C2—H2C	109.5	C10—C11—C12	122.7 (3)
H2A—C2—H2C	109.5	C10—C11—H11	118.7
H2B—C2—H2C	109.5	C12—C11—H11	118.7
C4—C3—N1	112.4 (3)	C7—C12—C11	116.5 (3)
C4—C3—H3A	109.1	C7—C12—Pd1	123.8 (2)
N1—C3—H3A	109.1	C11—C12—Pd1	119.7 (2)
C4—C3—H3B	109.1	F1—C13—F3	103.9 (3)
N1—C3—H3B	109.1	F1—C13—F2	102.2 (3)
H3A—C3—H3B	107.8	F3—C13—F2	104.2 (3)
C3—C4—N2	113.9 (3)	F1—C13—Pd1	118.3 (2)
C3—C4—H4A	108.8	F3—C13—Pd1	113.9 (3)
N2—C4—H4A	108.8	F2—C13—Pd1	112.8 (2)
C3—C4—H4B	108.8	C1—N1—C3	111.6 (3)
N2—C4—H4B	108.8	C1—N1—C2	106.8 (3)
H4A—C4—H4B	107.7	C3—N1—C2	107.2 (3)
N2—C5—H5A	109.5	C1—N1—Pd1	112.3 (2)
N2—C5—H5B	109.5	C3—N1—Pd1	106.2 (2)
H5A—C5—H5B	109.5	C2—N1—Pd1	112.7 (2)
N2—C5—H5C	109.5	C5—N2—C6	108.4 (3)
H5A—C5—H5C	109.5	C5—N2—C4	112.3 (3)
H5B—C5—H5C	109.5	C6—N2—C4	106.8 (3)
N2—C6—H6A	109.5	C5—N2—Pd1	113.6 (2)
N2—C6—H6B	109.5	C6—N2—Pd1	112.9 (2)
H6A—C6—H6B	109.5	C4—N2—Pd1	102.7 (2)
N2—C6—H6C	109.5	C12—Pd1—C13	86.65 (12)
H6A—C6—H6C	109.5	C12—Pd1—N1	93.25 (10)
H6B—C6—H6C	109.5	C13—Pd1—N1	177.08 (12)
C12—C7—C8	122.0 (3)	C12—Pd1—N2	173.23 (10)
C12—C7—H7	119.0	C13—Pd1—N2	97.25 (12)
C8—C7—H7	119.0	N1—Pd1—N2	83.14 (10)
			
N1—C3—C4—N2	−55.4 (5)	C11—C12—Pd1—N1	95.3 (2)
C12—C7—C8—C9	0.3 (5)	F1—C13—Pd1—C12	−40.6 (3)
C7—C8—C9—C10	1.0 (6)	F3—C13—Pd1—C12	81.9 (3)
C7—C8—C9—F4	179.4 (3)	F2—C13—Pd1—C12	−159.6 (3)
C8—C9—C10—C11	−0.3 (6)	F1—C13—Pd1—N2	145.0 (3)
F4—C9—C10—C11	−178.7 (3)	F3—C13—Pd1—N2	−92.6 (3)
C9—C10—C11—C12	−1.6 (5)	F2—C13—Pd1—N2	25.9 (3)
C8—C7—C12—C11	−2.1 (5)	C1—N1—Pd1—C12	−61.5 (2)
C8—C7—C12—Pd1	176.0 (2)	C3—N1—Pd1—C12	176.3 (2)
C10—C11—C12—C7	2.8 (5)	C2—N1—Pd1—C12	59.1 (2)
C10—C11—C12—Pd1	−175.4 (3)	C1—N1—Pd1—N2	112.8 (2)
C4—C3—N1—C1	−87.2 (4)	C3—N1—Pd1—N2	−9.5 (2)
C4—C3—N1—C2	156.3 (4)	C2—N1—Pd1—N2	−126.6 (2)
C4—C3—N1—Pd1	35.5 (4)	C5—N2—Pd1—C13	−71.4 (3)
C3—C4—N2—C5	−80.8 (4)	C6—N2—Pd1—C13	52.6 (3)
C3—C4—N2—C6	160.5 (4)	C4—N2—Pd1—C13	167.1 (3)
C3—C4—N2—Pd1	41.5 (4)	C5—N2—Pd1—N1	105.7 (3)
C7—C12—Pd1—C13	94.4 (3)	C6—N2—Pd1—N1	−130.4 (2)
C11—C12—Pd1—C13	−87.6 (3)	C4—N2—Pd1—N1	−15.8 (2)
C7—C12—Pd1—N1	−82.7 (3)		
